# Use of Repeated Within-Subject Measures to Assess Infants’ Preference for Similar Others

**DOI:** 10.3389/fpsyg.2019.02239

**Published:** 2019-10-03

**Authors:** Amir Cruz-Khalili, Katrina Bettencourt, Carolynn S. Kohn, Matthew P. Normand, Henry D. Schlinger

**Affiliations:** ^1^Department of Psychology, University of the Pacific, Stockton, CA, United States; ^2^Department of Psychology, California State University, Los Angeles, Los Angeles, CA, United States

**Keywords:** infants, social evaluation, methodological replication, preference, repeated measures, within-subject

## Abstract

Research employing single-choice paradigms in which an infant is asked to make a single choice between two puppets suggest that infants show a preference for prosocial others and those who are similar to themselves. However, the extent to which infants’ preference for similar others is stable is unknown, as are other factors within the paradigm that may influence infants’ choices. The purpose of this study (two experiments, *N* = 44 infants, aged 8–15 months) was to replicate and extend previous work by including (1) within-subject repeated measures and (2) an experimental manipulation of a plausible demand characteristic. Results for the first-choice trial indicated a majority of the infants did not choose the similar puppet. Results from the within-subject repeated trials also indicated that a majority of the infants did not choose the similar puppet but a majority did choose a puppet from the same side. The experimental manipulation of the demand characteristic showed no effect on infant puppet choices. These results suggest that a closer examination of the single-choice puppet paradigm for assessing infants’ social evaluation is warranted. These findings also support recommendations made by others, including publishing null findings, standardizing data collection and reporting methods, and examining individual differences by employing within-subject designs with repeated measures.

## Introduction

### Do Infants Prefer Similar Others? A Replication and Extension

Infants as young as 5 months (Hamlin et al., 2007, 2010, 2011; Hamlin and Wynn, 2011, 2012) and older infants and toddlers (Geraci and Surian, 2011; Buon et al., 2014; Scola et al., 2015; Woo et al., 2017; Chae and Song, 2018) seem capable of socially evaluating the behavior of others and appear to show a preference for prosocial others (for reviews, see Martin and Olson, 2015; Holvoet et al., 2016; Van de Vondervoort and Hamlin, 2018) as well as those who are similar to themselves along some dimension (e.g., Hamlin and Wynn, 2012; Mahajan and Wynn, 2012; Hamlin et al., 2013; Burns and Sommerville, 2014; Gerson et al., 2017). These findings have led researchers to hypothesize that we may be born with something akin to an innate moral core (Cook, 2013; Hamlin, 2013) or early strong tendencies (Martin and Olson, 2015; Holvoet et al., 2016; Hare, 2017), which include a preference for similar others (Hamlin and Wynn, 2012; Mahajan and Wynn, 2012; Hamlin et al., 2013) and that these tendencies observed during infancy may predict social and behavioral adjustment at 4 years of age (Tan et al., 2018).

Much of this literature is based on a methodology in which an infant is asked to make a single choice between two puppets (Martin and Olson, 2015; Holvoet et al., 2016; Margoni and Surian, 2018; Van de Vondervoort and Hamlin, 2018)^1^. In studies examining infants’ preference for prosocial agents, with a few exceptions (e.g., Scola et al., 2015), each infant sits on his or her parent’s lap and together they watch a puppet show during which a prosocial puppet helps a protagonist puppet and an antisocial puppet hinders this same protagonist puppet. After the puppet show, the helper and hinderer puppets are presented to the infant, who is then asked to make a single choice, with choice defined as the infant simultaneously looking at and reaching for one of the puppets. Researchers have modified this single-choice assessment paradigm to examine infants’ preference for similar others. For example, in their study, Mahajan and Wynn (2012) asked infants to choose between two foods, watch a puppet show in which one puppet stated a preference for one food and a dislike for the other food and the second puppet stated the opposite preference, and then make a single choice between the two puppets. Infants in the high salience experiment (*N* = 32) made their food choice first to test whether affiliative priming (Martin and Olson, 2015, p. 165) or increased “saliency” of the similarity between the infant’s food choice and the puppet’s food preference would affect infants’ choices. In the low salience experiment, infants (*N* = 16) made their food choice last. Results showed more infants in the high salience experiment (84%) chose the puppet that liked the same food compared to infants in the low salience experiment (44%). The authors offered these results as evidence that “Like older children and adults, even a minuscule non-arbitrary difference is sufficient to trigger a similarity bias” (p. 231) in preverbal infants.

Although it is possible infants prefer similar others, failed replications of studies examining infants’ preference for prosocial agents using a similar methodology (Scarf et al., 2012; Cowell and Decety, 2015; Salvadori et al., 2015; Holvoet et al., 2016; Nighbor et al., 2017) suggest several features of the experimental arrangement warrant closer attention. Many things happen in the staged scenario, and the putative similarity in food choice between puppet and infant is only one of them. Recent literature reviews (Martin and Olson, 2015; Holvoet et al., 2016; Van de Vondervoort and Hamlin, 2018) and a meta-analysis (Margoni and Surian, 2018) describe studies in which researchers using the same or very similar methods did not obtain similar results. Salvadori et al. (2015) directly replicated the methods of Hamlin and Wynn (2011) and found only 15 of the 24 infants (62.5%) selected the prosocial puppet. Even following subsequent procedural modifications suggested by Hamlin (Salvadori et al., 2015), only 12 of the 24 (50%) infants selected the prosocial puppet in their second experiment. However, 17 of the 24 (70.8%) infants selected the puppet presented on the right side, indicating that something other than the social aspect of the puppet show might direct infants’ choices. Cowell and Decety (2015) also replicated Hamlin et al.’s (2007) puppet paradigm and in their study only 54 (50%) of the infants chose the prosocial over the antisocial puppet; they did not report information about infants’ side choices.

As highlighted by these studies, independent researchers have obtained different results using similar methods to assess infants’ preferences via the single-choice puppet paradigm. One possibility is that these differences result from researcher degrees of freedom (Wakeley et al., 2000; Orne, 2009; Rosenthal, 2009; Simmons et al., 2011; Peterson, 2016; Eason et al., 2017), or “flexibility in data collection, analysis, and reporting” (Simmons et al., 2011, p. 1359) and other unintentional biases (Haith, 1998) that affect the likelihood researchers will observe significant experimental effects. Although many types of researcher degrees of freedom can contribute to these replication failures, two that seem particularly relevant to this line of research: single versus repeated within-subject assessments, and demand characteristics of the experimental situation.

## Repeated Within-Subject Assessments

A single measure of infant puppet choice may be insufficient to identify something like a preference for prosocial and similar others and might obscure the possibility that infants’ choices are directed by other factors. Repeated measures of each infant’s choice would seem to be an efficient method to address this limitation; however, we were able to locate only four published studies that used within-subject repeated measures (Hamlin and Wynn, 2012; Dahl et al., 2013; Gerson et al., 2017; Nighbor et al., 2017).

Hamlin and Wynn (2012) asked infants (*N* = 48, mean age 16 months) to choose between two bowls of food, which were identical except that one was filled with red Cheerios™ and the other with purple Cheerios™. Food preference was defined as the food the infant chose on more than two out of the four trials. During the food choice trials, 14 of the infants (29%) chose the same food four times and 14 infants (29%) chose each type of food exactly twice. Thus, several infants appeared to show no preference between the two foods. This is problematic because infants’ food choice served as the key measure on which puppet choice, and thus preference for similar others, was assessed.

Dahl et al. (2013) examined whether toddlers (*N* = 84, three groups, with mean ages of 17-, 22-, and 26 months) were more likely to exhibit helping behavior toward a prosocial or antisocial actor using two live actors across three or four trials; the toddlers viewed the show prior to the start of each helping trial. Out of the 84 toddlers, 43 (51%) helped either actor at least once. However, when assessed across three trials, this tendency diminished with 39% helping the prosocial actor on the first trial, 22% on the second trial, and 25% on the third trial. These numbers varied even more for the 17-month- and 22-month-old toddlers, where a total of 23% helped the prosocial actor on the first trial, 27.5% on the second trial, and 18% on the third trial. Dahl et al. (2013) noted that their results illustrate the importance of reporting within-subject repeated measures.

Nighbor et al. (2017) replicated Hamlin and Wynn’s (2011) puppet paradigm and extended the methodology by having infants make four additional choices. Thirteen of the 20 infants (65%) chose the helper puppet on the first trial. However, when all five choices were examined, on at least four of the five trials, 50% of the infants reached for the same side; whereas, 20% chose the prosocial puppet and 20% chose the antisocial puppet, suggesting some infants show a strong within session preference for a particular side (e.g., Diedrich et al., 2001; Fisher-Thompson and Peterson, 2004; Woo et al., 2017).

Using a slightly altered version of the single-choice puppet paradigm, Gerson et al. (2017) asked infants to make five choices between two toys. Their results showed that infants who observed a puppet choose the toy were somewhat more likely to choose that same toy (67%) compared to infants who observed the puppet be assigned the toy (52%). Infants’ side choices were not reported, and the experimenter who conducted the familiarization and choice trials was not blind to the puppet assignments or the study hypothesis.

In addition to the presence of perseverative side reaching (e.g., Salvadori et al., 2015; Nighbor et al., 2017; Woo et al., 2017), Diedrich et al. (2001) observed that “infants perseverated when reaching for two identical targets, but infants made non-perseverative responses when reaching in the presence of a highly distinctive second target” (p. 263). Notably, the puppet paradigm used to assess infant preference for prosocial others and similar others typically makes use of two identical puppets that differ only by the color of the shirts they are wearing. Use of repeated measures would help identify if infants are able to discriminate between identical puppets wearing different colored t-shirts, or if this method inadvertently encourages side perseveration.

With the few exceptions described above, no studies have used repeated choice measures to assess infant preference, even though there is a substantial body of literature on the use of preference assessments, including those for typically developing toddlers (Cote et al., 2007), individuals who cannot otherwise communicate their preferences (Kang et al., 2013), and nonhuman animals (Cox et al., 1996; Vicars et al., 2014), all of which assess the individual’s response across multiple trials using within-subject counterbalancing of items. Although preferences can change over time (Hanley et al., 2006; Kang et al., 2013), individuals generally select items deemed preferred more frequently than less preferred items within a single session, yielding a hierarchy of most to least preferred (Hanley et al., 2006; Kang et al., 2013). Taken together, these studies highlight the importance of examining individual differences (e.g., Holvoet et al., 2016) through the use of within-subject repeated measures when assessing infants’ preferences using the single-choice puppet paradigm.

## Demand Characteristics

Although repeated measures might address one important source of response variation, other factors also should be investigated. For example, Margoni and Surian (2018) compared the effect sizes from one specific group of researchers who conducted over half of all published studies to the rest of the published studies and found that studies published by that specific group of researchers had significantly larger effect sizes. One plausible explanation for the larger effect sizes coming from one group of researchers may have to do with demand characteristics.

In one of the first studies published using the puppet paradigm, Hamlin et al. (2007) had infants and their parents watch a show depicting one prosocial and one antisocial puppet and then asked infants to choose between the prosocial and antisocial puppets. In this first study, 100% of the 6-month-old infants (*n* = 12) and 87.5% of the 10-month-olds (*n* = 16) chose the prosocial puppet; however, parents were not asked to close their eyes during the puppet show or puppet choice measure. Results of this magnitude have not been replicated in subsequent studies in which parents were blind (i.e., asked to turn away or close their eyes) to the puppet choice task, with results typically showing between 60 and 72% (*M* = 64%) of the infants choosing the prosocial puppet (Margoni and Surian, 2018). For example, by definition, parents in Mahajan and Wynn’s (2012) high-salience group were not blind to key variables (i.e., infant food choice and infant puppet choice) and 84% of these infants chose the similar puppet. However, parents in the low salience group were blind to their infants’ food choice (i.e., because it came after the infants’ puppet choice) and 44% of these infants chose the similar puppet. This type of arrangement, whereby the parent observes their infant choose a food prior to choosing a (similar) puppet may result in demand characteristics or subtle variations in the behavior of the parent or experimenter which influences the infant’s choice (Wakeley et al., 2000; Orne, 2009; Rosenthal, 2009; Eason et al., 2017).

An additional, although perhaps subtler, form of demand characteristic that might contribute to the larger effect size (Margoni and Surian, 2018) is the information parents are exposed to prior to participating in the study. Websites advertising and describing Hamlin, Wynn, and colleagues’ research (e.g., https://campuspress.yale.edu/infantlab/our-studies/; http://cic.psych.ubc.ca; http://cic.psych.ubc.ca/2018/10/29/welcome-to-the-centre-for-infant-cognition/) provide substantial information and materials directly relevant to their studies. Parents motivated to volunteer for little or no monetary compensation (websites and papers do not list monetary compensation) may also be sufficiently interested in the research to read about it prior to participating in the studies. Parents’ pre-study access to this information could alter their behavior in measurable ways which might then affect their infant’s behavior (e.g., Rosenthal and Jacobson, 1968; Orne, 2009; Peterson, 2016).

## The Current Study

Calls have been made for independent replications of the methods used to examine infants’ social preferences (e.g., Martin and Olson, 2015; Peterson, 2016; Margoni and Surian, 2018) along with extensions to address specific concerns (Holvoet et al., 2016; Hinten et al., 2018). The purpose of the current study was to replicate and extend this line of research. We chose to replicate Mahajan and Wynn’s (2012) high- and low-salience group methodology. We then extended this research in two important ways by including (1) within-subject repeated measures and (2) an experimental manipulation of two plausible demand characteristics.

## General Method

### Overview

**Figure 1:**
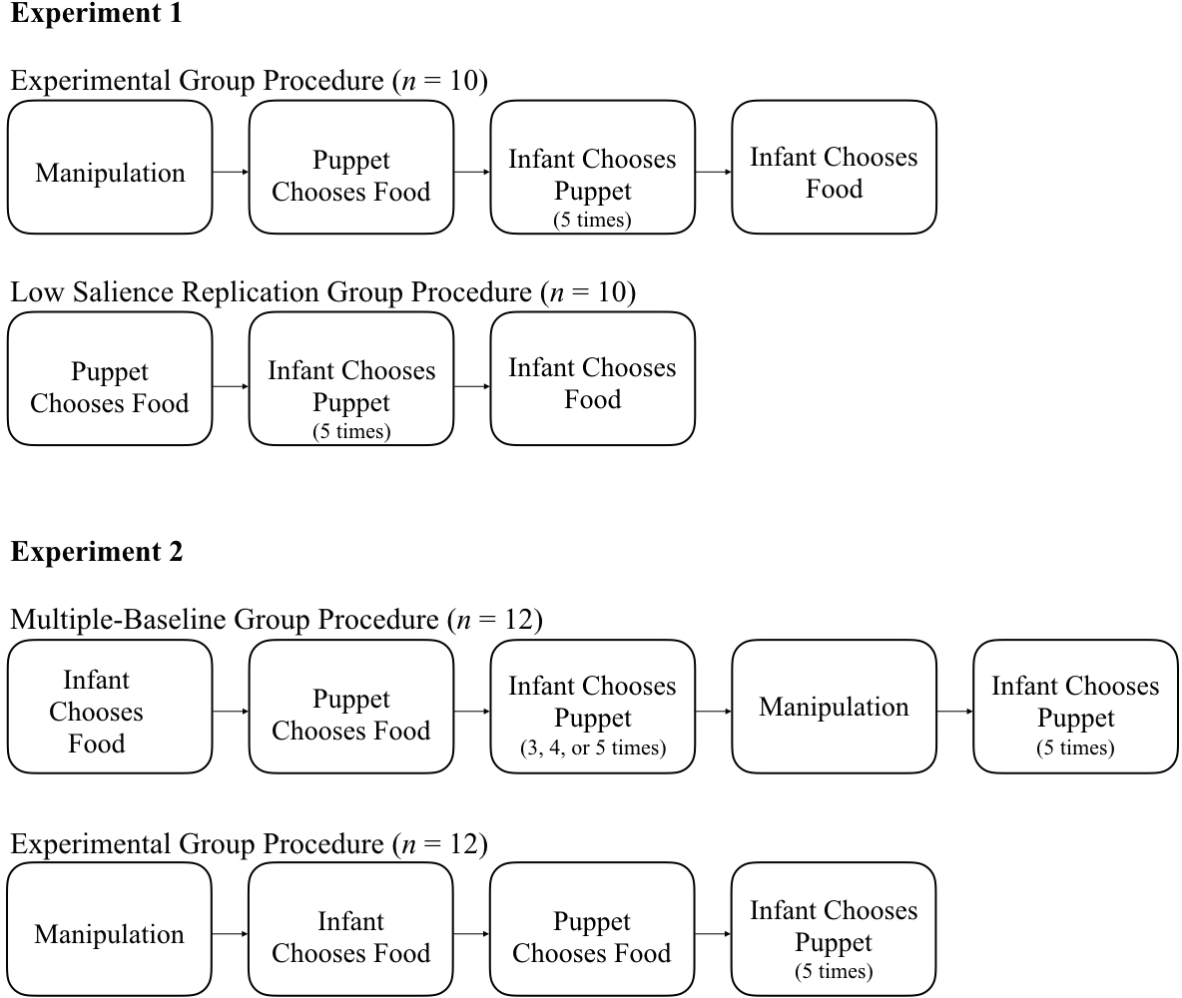
General experimental procedure for each group in Experiment 1 **(top)** and Experiment 2 **(bottom)**.

Experiments 1 and 2 were based on the methods reported by Mahajan and Wynn (2012) and depicted in their supplementary videos^2^ and included three components: the Infant-Chooses-Food task, the Puppet-Chooses-Food puppet show, and the Infant-Chooses-Puppet task, each of which are described below under Section “Procedure.” Only the order of the tasks differed in each experiment. Figure 1 depicts the procedures for Experiment 1 (top half) and Experiment 2 (bottom half). As recommended (e.g., Simmons et al., 2011; Oakes, 2017), sample size was determined prior to the start of data collection.

### Participants

In total, participants were 44 infant-parent dyads. Specific demographic information about infants and their parents is provided under Section “Methods” of each experiment. This study was carried out in accordance with the principles of the United States Department of Health and Human Services, Office for Human Research Protections, Federal Policy for the Protection of Human Subjects (often referred to as the Common Rule). The protocol was approved by the University of the Pacific institutional review board prior to recruitment and data collection. All parents reviewed and signed informed consent and video recording consent forms prior to their participation.

### Setting and Materials

All sessions lasted 15–20 min and took place in a room on the university campus or a room in the parent’s home devoid of distracting stimuli (i.e., toys, music, other noise, or people). When meeting in a room in the parent’s home (12 of 20 sessions in Experiment 1 and 22 of 24 sessions in Experiment 2), researchers concealed or removed all potentially distracting items from the room and from view of the infant and his or her parent.

Materials included one lamb puppet wearing a yellow shirt, one lamb puppet wearing an orange shirt, two transparent plastic bowls, and two snack foods: graham crackers and green beans^3^. Canned green beans were used with the first five participants in Experiment 1; however, beginning with the sixth participant and for all participants in Experiment 2, we switched to dehydrated green beans to avoid vegetable decay.

### Procedure

During the initial phone contact, and again prior to starting the experimental sessions, we asked parents if their infants had allergies to green beans or the ingredients in graham crackers; no parents reported their infants had allergies to either food. Following completion of the study session, parents were compensated for their time with a $10 gift card (Experiment 1) or a $20 gift card (Experiment 2) to a retail store of their choice (e.g., Target, Amazon.com); three parents in Experiment 1 chose to receive a $10 baby-clothing item from Target in lieu of a gift card.

For both experiments, we used within- and between-subjects designs. Each experiment had two groups and infant-parent dyads were randomly assigned to one of the two groups. All infant-parent dyads were exposed to the same general procedures described below. Key differences between experiments and groups involved the order of the tasks, the order and type of experimental manipulation of the demand characteristic, and the number of times infants were asked to choose a puppet.

#### Puppet-Chooses-Food Puppet Show

Each infant was seated on his or her parent’s lap on one side of a table, opposite Experimenter 1 (E1, primary data collector), Experimenter 2 (E2, puppeteer), and Experimenter 3 (E3, secondary data collector). Before beginning the puppet show, E3 set up the video camera to face E2 to record the puppet shows for later integrity checks. E1 and E3 then left the room before E2 presented the puppet show to the infant and parent using a script. As described by Mahajan and Wynn (2012), the puppeteer (E2) was visible to both the infants and the parents during all puppet shows. For all participants, E2 held each puppet equidistant behind the food. During the puppet show, one puppet verbally stated a preference for one food and a dislike for the other food while the other puppet stated the opposite preference. To indicate the puppet was “tasting” the food, E2 placed the puppet’s face into the bowl, made eating noises, then lifted the puppet’s head so it was facing the infant and said either, “Mmmm, yum. I like that” in a high-pitched voice or “Ewww, yuck. I don’t like that” in a low-pitched voice. E2 then returned the puppet to its previous position, and then with the other puppet, repeated the scenario with the same verbal statements but for the opposite foods. Puppet side, puppet color, and order of the puppets’ expression of food preference were counterbalanced between subjects. These procedures replicated those described by Mahajan and Wynn (2012) and depicted in their supplementary videos (see text footnote 2), with two exceptions. First, there was a discrepancy in the description of the puppet show scripts between the text in the manuscript and the supplementary videos. The manuscript described the experimenter as stating “…I like that” or “…I don’t like that” during the puppet show, whereas the video showed the experimenter clearly stating the names of the foods. We followed the published methods rather than the video. Second, Mahajan and Wynn (2012) did not describe, either in their manuscript or in the supplementary video, how infants were prompted to make either a puppet choice or a food choice; however, Hamlin et al. (2013) provided such descriptions and a video for a very similar infant choice task in which the experimenter asked infants “Which one do you like?”, so we used that prompt in the current study.

#### Infant-Chooses-Puppet Task

After the puppet show, the food bowls were removed, E2 turned away or left the room and E1 and E3 returned. E1, blind to the puppet show and puppets’ food choices, presented the two puppets to the infant by leaning toward the infant and saying, “Hi,” then shook the puppet in their right hand while saying, “Look.” The same action was used for the puppet in the experimenter’s left hand. Next, E1 said, “Hi,” once again before presenting the two puppets equidistant and within reach of the infant. E1 asked, “Which one do you like?” (Hamlin et al., 2013). Infant choice was recorded by E1 and E3 as the first puppet the infant concurrently looked at and touched (Mahajan and Wynn, 2012). E1 then asked the infants to make four additional puppet choices, using the same procedure of saying “Hi,” saying “Look,” and then offering the puppets. The side on which the puppets were presented was counterbalanced within-subject prior to each choice made by the infant. When infants did not make a choice right away, E1 shook both puppets and repeated the phrase “Which one do you like?” If 10 s passed and no choice was made, E1 repeated the phrase one more time. If the infant still did not make a choice, E1 marked the data sheet “no choice,” switched the puppets, and moved on to the next choice on the data sheet. Depending on their group assignment, this process was repeated until infants made at least five choices; differences in the number of puppet choices are described separately under the specific Method sections for each experiment.

#### Infant-Chooses-Food Task

Two clear bowls, one containing graham crackers and the other containing green beans, were placed on each end of the stage front. Mahajan and Wynn (2012) did not describe counterbalancing the bowls of food, but in their supplementary videos, the graham crackers were always on infant’s left side; therefore, we always placed graham crackers on the infants’ left during the puppet show and infant food choice task, with three exceptions: once during Experiment 1 and twice during Experiment 2 graham crackers were mistakenly placed on the participants’ right side (P1 in Experiment 1 and P12 and P13 in Experiment 2). E1 presented the infant with the two bowls of food and asked, “Which one do you like?” and moved each bowl a little closer but equidistant to the infant (Hamlin et al., 2013). E1 and E3 recorded the infant’s choice as the first food the infant picked up (Mahajan and Wynn, 2012).

#### Post-study Questionnaire

Upon completion of the study, each parent was asked to provide a written response to the question, “What do you think the puppet show was about?” Prior to reviewing the completed surveys, experimenters composed lists of words they believed would indicate the parent understood the purpose of the puppet show or the study (see Table 1 for the wordlist). Parents were said to correctly identify the purpose of the puppet show if they used words such as: food preference, similar, same, puppet, or liked. Parents were said to have correctly identified the key independent variables in the experiment, but not necessarily the content of the puppet show, if they wrote words such as: parent or parental influence or bias or side bias. All of these words were coded in context, meaning that the adjacent words had to be related to the study or puppet show. Parents’ answers to the question were coded at the conclusion of the study. One coder (Experiment 1) or two coders (Experiment 2) independently rated each parent response as either reflecting or not reflecting the purpose of the study; for Experiment 2, agreement between the two coders was 100%.

**Table 1:** Abbreviated list including only those words used to code parents’ correct responses to the post-study questionnaire in Experiment 1 and Experiment 2.

Correctly identified purpose	
Parent influence	Right
Parent bias	Left
Ingroup bias	Side bias
Racism	Puppet-baby association
Prejudice	Article influence parent/infant behavior^*^
Saliency	

Use of the above terms in response to the question, “What do you think the puppet show was about?” constituted either correct identification of the purpose or identification of the key variables. The phrase marked with an asterisk (*) was only coded for Experiment 2.

### Interrater Reliability

Prior to analyzing infants’ choices, we assessed the reliability of our coding of infants’ choices. For both experiments, E1 and E3 independently recorded the infants’ in-session food choice and puppet choices for 100% of the sessions. After all participants completed the study, another coder (E2) coded all infants’ choices in 100% of the video recorded sessions. Interrater reliability (IRR) was calculated as the number of agreements divided by the number of agreements plus disagreements multiplied by 100.

For all sessions in Experiment 1, in-session IRR for puppet choices was 100%. All sessions were video recorded, and 35% of the videos were randomly chosen to be recoded for IRR purposes; video IRR was 90%. For data analyses, we used in-session data because in-session IRR was 100%. For Experiment 2, in-session IRR was 100% for food choice and infant puppet choices and 98% (range, 88–100%) for infant side choices. IRR between E1’s live coding and the video coder and between E3 and the video coder was 89% (range of 72–100%) for infants’ puppet choices and side choices. After reviewing the two videos with the discrepant codes, we noted that the primary data collector (E1) and video coder’s data were correct and in agreement; it was the live coder that incorrectly coded the infant’s choices, and the discrepancies were resolved.

### Data Analyses

We used two-tailed binomial tests to examine the probability of results for infants’ first choice. We used visual analyses and descriptive statistics to examine the repeated measures of infant puppet choice and infants’ single food choice.

## Experiment 1

### Methods

#### Participants

Participants were 20 infants, 8–15 months old (*M* = 10 months, 18 days) and their parents. Fifty percent of the infants were Caucasian, 40% mixed ethnicity, 5% Hispanic, and 5% other. Parent participants were 50% Caucasian, 20% Hispanic, 20% mixed ethnicity, 5% Black/African-American, and 5% other. Fifteen percent of the parents held advanced degrees, 40% graduated college, and 45% had some college education.

#### Design and Procedure

Figure 1 (top half) depicts the specific procedures. Half of the infant-parent dyads (*n* = 10) were assigned to the Control Group, a direct replication of Mahajan and Wynn’s (2012) low salience condition in which the infants viewed the puppet show and then chose a food. The other half of the parent-infant dyads (*n* = 10) were assigned to the Experimental Group (described in the next section). Infants in both groups were asked to choose a puppet five times.

#### Experimental Manipulation

The Experimental Group followed the same procedure as the Control Group with one exception: the inclusion of a demand characteristic. After the parent signed the consent forms and completed the demographics questionnaire but before the puppet show, E1 said, “At this time we would like your baby to make a private food choice. I am going to ask that you and the other experimenters close their eyes while I present [infant’s name] with two foods.” E2, E3, and the parent closed their eyes and E1 made “rustling” noises then circled an answer on a data sheet. No actual foods were presented to the infant during the private food choice and the parents were always told their infant chose graham crackers. This took approximately 10 s. E1 then said, “Okay, [Mom/Dad], you can open your eyes. On this data sheet, I circled the food [infant’s name] chose. I ask that you do not say it out loud because the other experimenters cannot know what your baby chose.” E1 showed the parent the data sheet reading “graham crackers” and “green beans” where “graham crackers” was always the circled choice. Once the parent saw the data sheet, it was put away, and the procedure continued as described in the previous sections (i.e., Puppet-Chooses-Food puppet show followed by Infant-Chooses-Puppet and Infant-Chooses-Food tasks).

This experimental manipulation was designed to approximate the high salience condition in Mahajan and Wynn (2012), during which parents observed their infants make a food choice prior to making a puppet choice, while ensuring that the infants did not choose a food prior to making a puppet choice. Thus, if more infants in this condition choose the similar puppet, it would not be due to the saliency of the similarity, as the infants were experiencing a low salience condition; the choice could instead be attributed to the parents knowing which food their child chose (e.g., a type of demand characteristic).

### Results and Discussion

#### First Puppet Choice

In the Experimental Group, three infants (30%) chose the puppet that preferred graham crackers (i.e., the similar puppet); these same infants also chose graham crackers during the food choice. This means seven infants (70%) in the Experimental Group chose the dissimilar puppet, *p* = 0.117, binomial test, two-tailed. When results were examined based on infants’ actual food choice (i.e., and not the manipulation), five infants (50%) selected the similar puppet (i.e., the same three infants who chose the graham cracker puppet and two infants who chose green beans and the puppet that liked green beans), *p* = 0.246, binomial test, two-tailed. In the Control Group, six infants (60%) chose the similar puppet, *p* = 0.205, binomial test, two-tailed.

#### First Choice Based on Side

In the Control Group, six infants (60%) chose a puppet on the right side on the first-choice trial, *p* = 0.205, binomial test, two-tailed. In the Experimental Group, three infants (30%) chose a puppet on the right side and seven infants (70%) chose a puppet on the left side, *p* = 0.117, binomial test, two-tailed.

#### Within Subject Stability of Puppet Choices Across Repeated Trials

**Figure 2:**
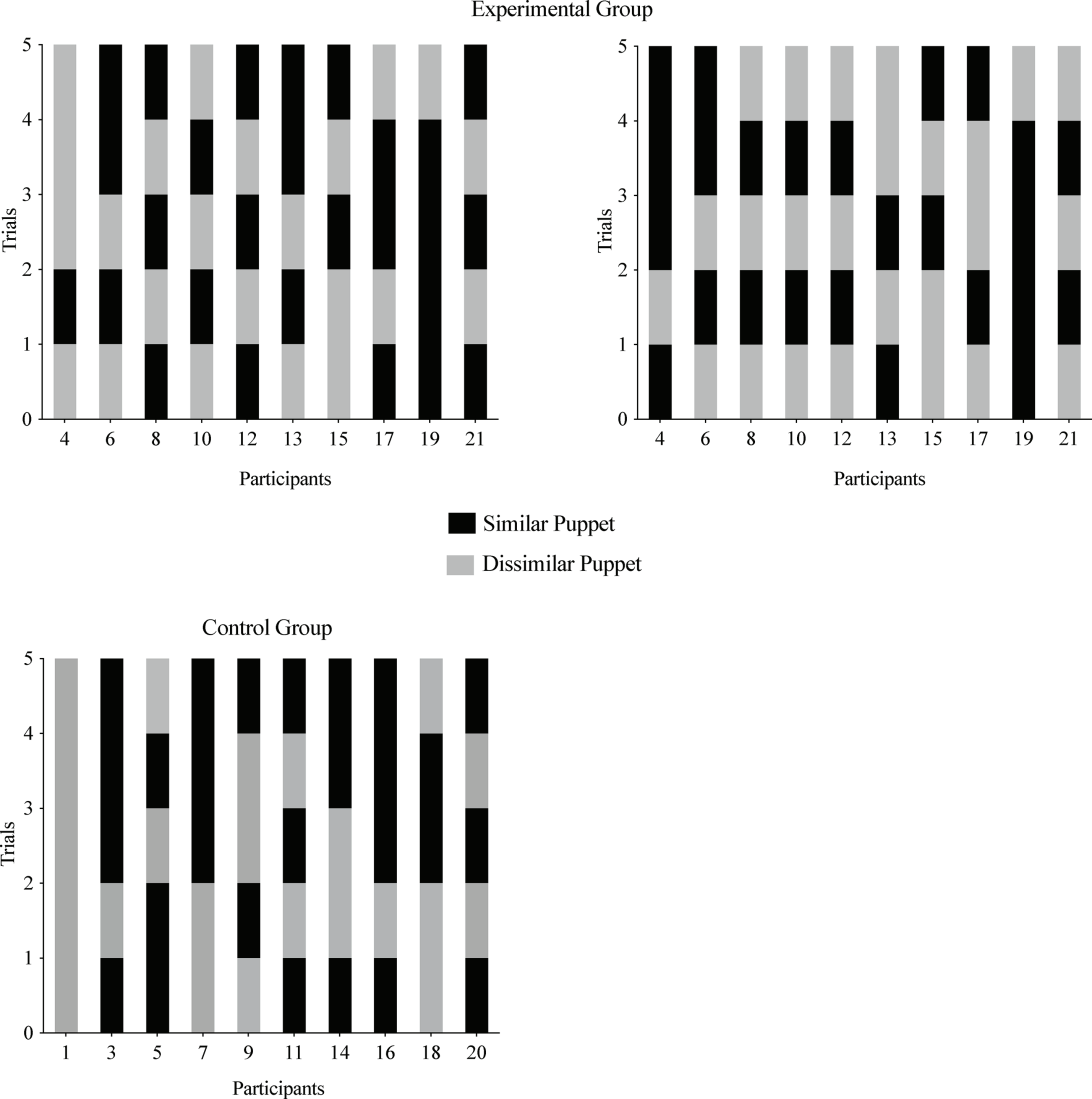
Experiment 1, infants’ choices of the similar puppet (black bar) or dissimilar puppet (gray bar) across the repeated choice trials. Infants are listed by participant number on the *x* axis, and each infant’s choice is depicted on the *y* axis. The top two graphs depict the Experimental Group choices. In the top left graph, the similar puppet is defined as the puppet that stated it liked the same food the infant chose during the food choice task. In the top right graph, the similar puppet is defined as the puppet that stated it liked graham crackers (i.e., the food parents were told their infants chose during the manipulation). The bottom graph depicts the Low Salience Group choices.

Figure 2 depicts results of the infants’ puppet choices across all five trials. In the Experimental Group, the similar puppet could be defined in two different ways. When the similar puppet was defined as the puppet who chose graham crackers (i.e., what the parent was told during the manipulation), two infants (20%) chose the similar puppet on at least 80% of trials. When the similar puppet was defined as the puppet who chose the same food as the infant, one infant (10%) chose the similar puppet on at least 80% of trials. In the Control Group, two infants (20%) chose the similar puppet on at least four trials. One infant from each group chose the dissimilar puppet on at least four trials.

#### Within Subject Stability of Side Choices Across Repeated Trials

**Figure 3:**
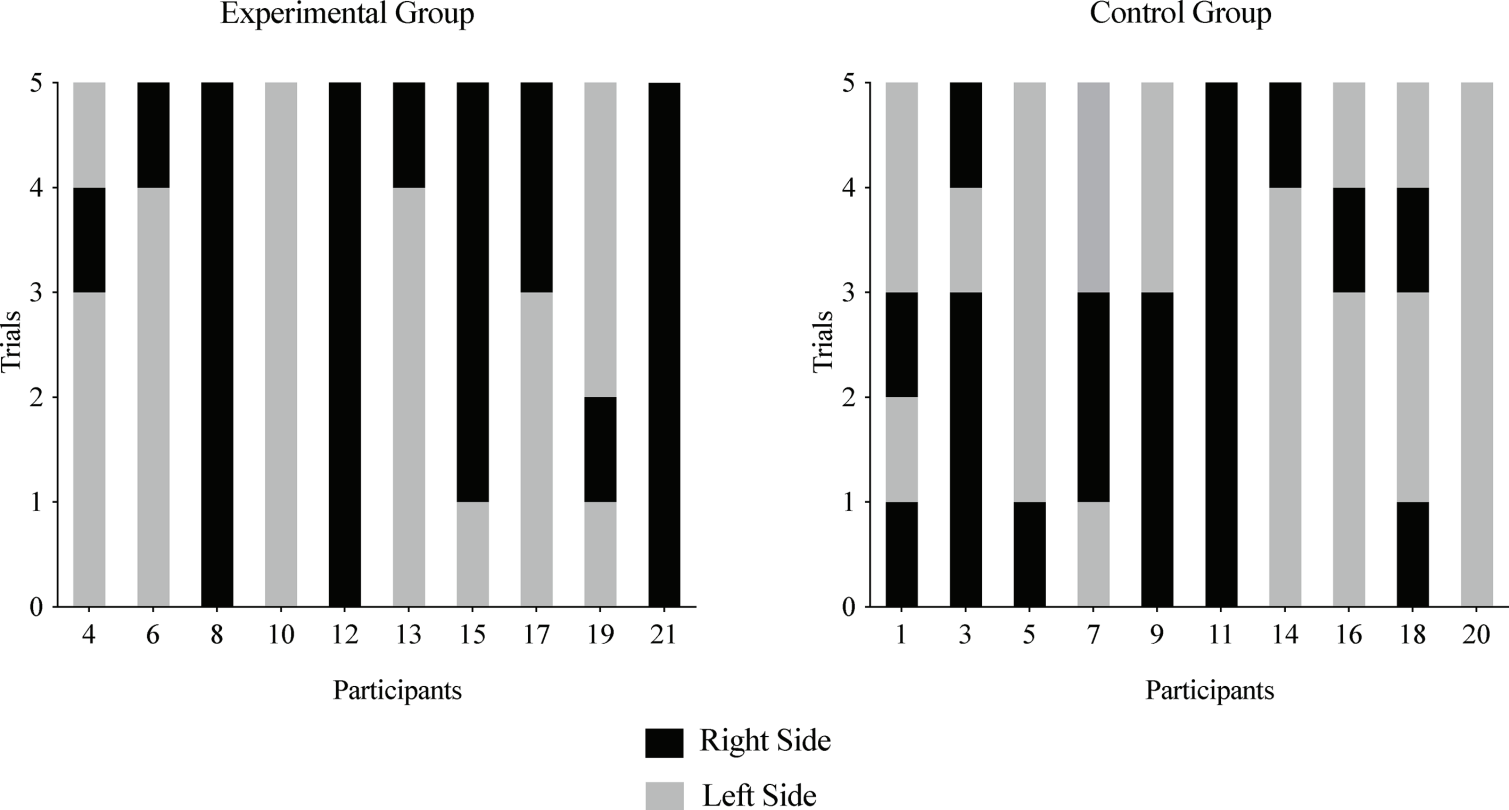
Experiment 1, infants’ choices of the puppet on the right side (black bar) or left side (gray bar) across repeated choice trials.

Figure 3 depicts results of the infants’ side choices across all five trials. Nine infants (90%) in the Experimental Group, *p* = 0.021, binomial test, two-tailed, and six infants (60%) in the Control Group, *p* = 0.754, binomial test, two-tailed, chose puppets presented on the same side on four or more trials. Altogether, 15 of the 20 (75%) infants selected a puppet on the same side (either right or left) on at least four trials, *p* = 0.041, binomial test, two-tailed.

#### Food Choice

During the food choice, six infants (60%) in the Experimental Group and seven infants (70%) in the Control Group chose green beans, *p* = 0.074, binomial test, two-tailed. Because green beans were always presented on the infants’ right side (with the one exception for P1 noted in the Method section, and P1 did not show side stability), we examined the potential relationship between side choice, puppet choice, and food choice. Of the 13 infants who chose green beans, five (38.5%) chose a puppet presented on the right on at least four trials.

#### Post-study Questionnaire

Upon completion of the experiment and all IRR calculations, we coded parents’ answers to the following question: “What do you think the puppet show was about?”, as indicating they either did or did not identify the purpose of the study. Based on the content words (see Table 1), six parents (30%) correctly described the purpose of the puppet show; two were from the Experimental Group. Four parents incorrectly identified the purpose of the puppet show but correctly identified the key independent variables under examination (e.g., side bias, parental influence); two were from the Experimental Group. The remaining 10 parents were unable to identify the purpose of the puppet show or the experiment (e.g., parent wrote the puppet show was about making healthy food choices).

### Summary

Overall, 45–55% of the infants chose the similar puppet on the first trial and 35–40% chose the same puppet on at least four of the five choice trials. However, 60% of the infants in the Experimental Group reached for the right side and 70% of the infants in the Control Group reached for the left side on their first choice-trial. On at least four of five trials, 10–20% of infants chose the similar puppet and 65% of the infants chose a puppet from the same side. More infants (65%) chose green beans in the current study compared to infants (25%) described by Mahajan and Wynn (2012).

## Experiment 2

### Methods

#### Participants

Participants were initially 26 infant-parent dyads recruited by word of mouth who lived in Northern California and Oregon. Two parent-infant dyads were excluded from the final sample because one infant exceeded the maximum age (i.e., was 18 months old) and one would not remain seated in her mother’s lap during the experimental session. Thus, the final sample consisted of 24 infant-parent dyads. Infants were aged 9–15 months (*M* = 11 months, 3 days). Seventy-five percent of the infants were Caucasian, 8% Hispanic, and 17% mixed or other ethnicities. Forty-two percent of the parents held advanced degrees, 25% graduated college, 25% had some college education, and 8% had a high school diploma.

#### Design and Procedure

All infant-parent dyads were randomly assigned to one of the two groups. Dyads in both groups completed the same tasks and were exposed to the experimental manipulation; the differences were the number of puppet choices infants made and the timing of the experimental manipulation (see Figure 1, bottom half).

Half of the infant-parent dyads (*n* = 12) were assigned to the Replication and Extension Group, designed to be a direct replication of Mahajan and Wynn’s (2012) high salience methodology with an extension. The extension consisted of a within-subjects multiple baseline design (Kazdin, 2010) in which infants made three to five puppet choices, then their parents were exposed to the experimental manipulation (described below), and then infants made an additional five puppet choices. The other half of the infant-parent dyads (*n* = 12) were assigned to the Experimental Group, designed to allow for a between-group comparison of infant puppet choice before (Replication and Extension Group) and after (Experimental Group) parents’ exposure to the manipulation. By designing the groups in this way, we were able to conduct group comparisons mirroring those of Mahajan and Wynn as well as a within-subjects comparison with half of the participants (Group 1) serving as their own controls (Kazdin, 2010).

#### Experimental Manipulation

The experimental manipulation was given to parents in both groups; only the timing differed. E1 asked parents to read a one-page document containing two brief paragraphs and a few pictures (e.g., teachers working with children) and explained that the document provided some description of the purpose of the study. Parents were given as much time as needed to review the document, usually 3–5 min. All information in the document was taken directly from the Yale Infant Cognition Center website^4^ which is available to the general public. The first paragraph described the purpose of the study and was taken from the website’s *Frequently Asked Questions* page^5^ and read as follows:

What is the purpose of this research? The purpose of our research is to learn more about the development of young infants and their early knowledge about the world. We are interested in how babies think and reason about their surrounding environment during their early months of life.

The second paragraph provided a brief description of the study and was taken from the website’s *Our Studies* page^6^ and read as follows:

Ingroups and outgroups. These studies ask whether infants, like adults, prefer those who are like them in some way versus those who are not like them. Using various cues to group membership (food preferences, clothing, toy preferences) babies are shown puppets who share these traits with them, and those who do not. We then see if babies prefer to play with a puppet who is like them.

We hypothesized this might serve as a relevant, accessible, and salient informational piece, as the website is available to all parents considering participation in studies conducted by the Yale Infant Cognition Lab, and may unintentionally influence parents’ behavior, which in turn may affect infant behavior.

After parents indicated they finished reading the document, E1 asked parents to rate three statements as true or false. These statements served as a manipulation check to identify whether parents read the document and were: (1) one purpose of research such as this is to learn more about how babies think and reason about their surrounding environment; (2) the purpose of this study is to determine if infants, like adults, prefer those who are more similar to themselves; and (3) babies who chose puppets who showed the same traits (i.e., food, clothing, or toy preferences) may be showing their preference toward puppets who are more like them. All three statements were true. Researchers scored all questions after the session ended.

#### Post-study Questionnaire

Based on our experience with Experiment 1, we developed three questions beyond the single question described under Section “General Method.” Two questions were added to examine whether parents were able to correctly identify the similar puppet: “During the study, which food did your infant choose?” followed by three options: (1) green beans, (2) graham crackers, and (3) do not remember; and, “During the puppet show, which puppet chose the same food your infant chose?”, followed by three options: (1) orange shirted puppet, (2) yellow shirted puppet, and (3) do not remember. Next, because four parents in Experiment 1 spontaneously told experimenters their infants had no prior exposure to the study foods, we added: “Please indicate how often your infant consumes the following food items” using a 4-point Likert scale (never, rarely, sometimes, or often). If infants have no or very little experience with the study foods, it is unlikely their food choice is indicative of preference and thus by extension, infants’ puppet choices are unlikely to indicate a preference for a similar other.

### Results and Discussion

#### First Puppet Choice

In the Replication and Extension Group, six of the 12 infants (50%) selected the similar puppet, *p* = 0.225, binomial test, two-tailed. In the Experimental Group, two of the 12 infants (17%) selected the similar puppet, meaning that 10 of the 12 infants (83%) selected the dissimilar puppet, *p* = 0.016, binomial test, two-tailed.

#### First Choice Based on Side

In the Replication and Extension Group, five infants (42%) chose a puppet on the right side and seven infants (58%) chose a puppet on the left side, *p* = 0.193, binomial test, two-tailed. In the Experimental Group, eight infants (67%) chose a puppet on the right side, *p* = 0.121, binomial test, two-tailed.

#### Within Subject Stability of Puppet and Side Choices Across Repeated Trials

##### Replication and Extension Group: Baseline (Pre-manipulation) Phase

**Figure 4:**
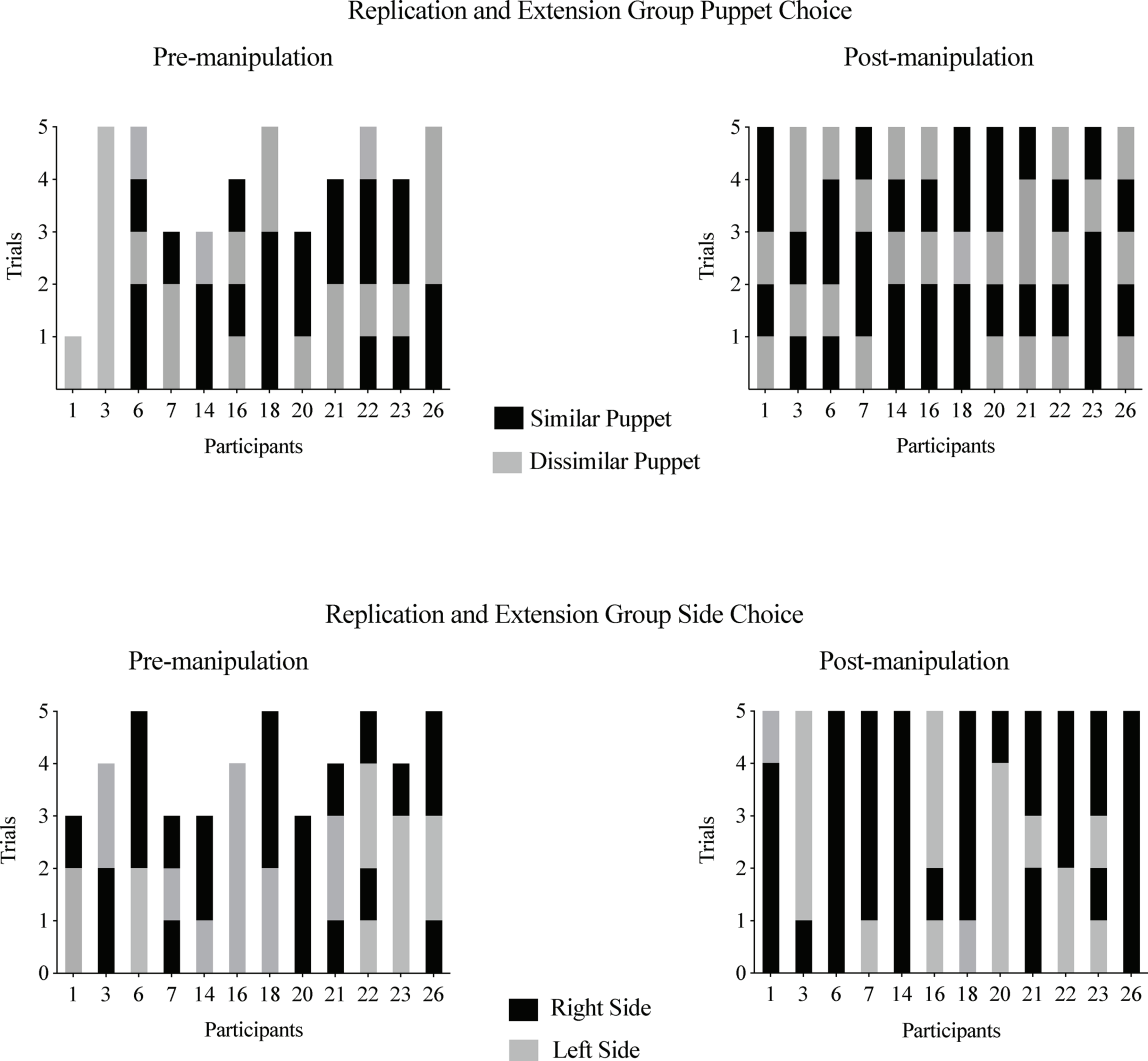
Experiment 2, infants’ choices across the repeated choice trials in the Replication and Extension Group. Infants are listed by participant number on the *x* axis, and each infant’s choice is depicted on the *y* axis. The left graphs depict infants’ selections prior to parents reading the article (i.e., baseline or pre-manipulation phase). The right graphs depict infants’ selections after parents read the article (i.e., intervention or post-manipulation phase). The top two graphs depict each infant’s choice of the similar puppet (black bar) or the dissimilar puppet (gray bar). The similar puppet is defined as the puppet that stated it liked the same food the infant chose during the food choice task; the dissimilar puppet is defined as the puppet that stated it disliked the same food the infant chose during the food choice task. The bottom two graphs depict infants’ choices of the puppet on the right side (black bar) or left side (gray bar) across repeated choice trials.

Based on their assignment in the multiple baseline design, infants made three, four, or five puppet choices after watching the puppet show but prior to their parents being exposed to the experimental manipulation. No clear group patterns were detected for either puppet selection (Figure 4, top left graph) or the side on which a puppet was presented (Figure 4, top right graph).

##### Replication and Extension Group: Post-manipulation Phase

After parents were exposed to the manipulation, on at least 80% of trials, two infants (17%) chose the similar puppet and no infants chose the dissimilar puppet (see Figure 4, top right graph). When side was examined, on at least 80% of trials, 10 infants (83%) chose a puppet on the same side, with 7 of these 10 infants choosing on the right side (see Figure 4, bottom right graph).

##### Replication and Extension Group: Pre- and Post-manipulation Phases

On at least 80% of all trials, one infant (8%) chose the similar puppet (also the only infant to choose the same puppet) and five infants (42%) chose a puppet from the same side.

##### Experimental Group

**Figure 5:**
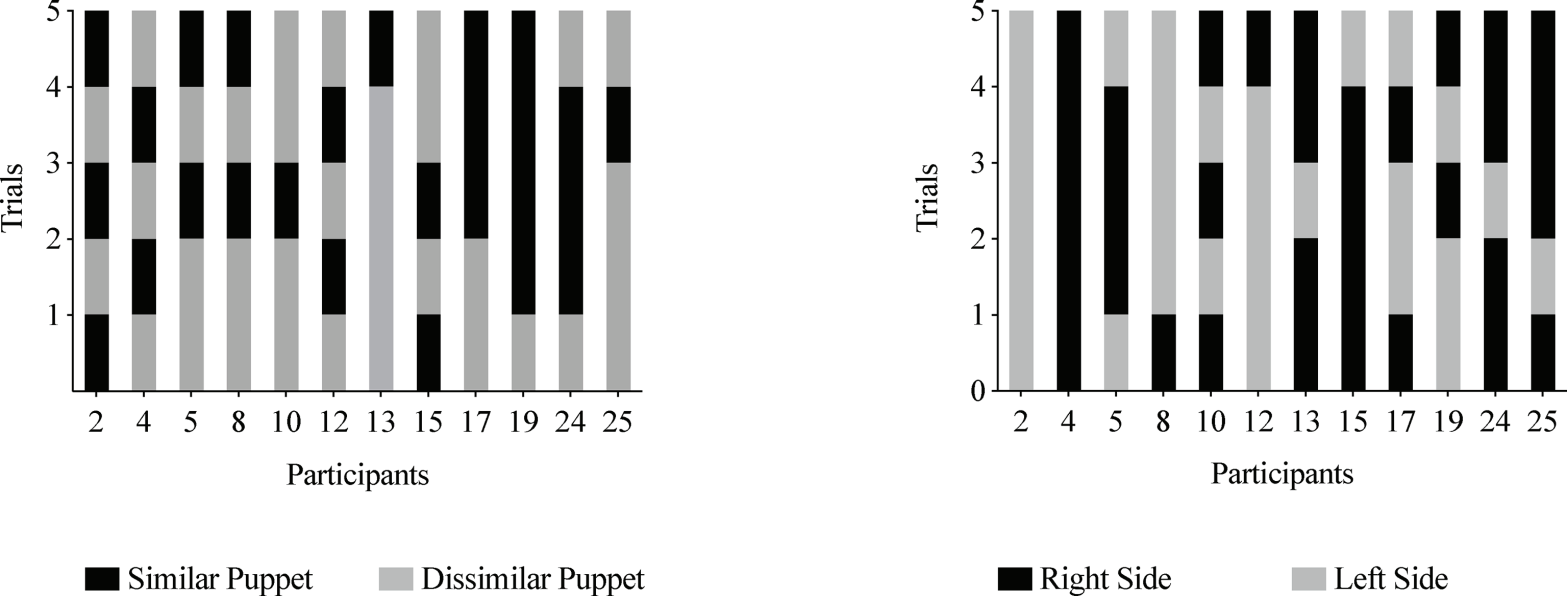
Experiment 2, Experimental Group puppet choices **(left panel)** and side choices **(right panel)**. Infants are listed by participant number on the *x* axis, and each infant’s choice is depicted on the *y* axis. The left graph depicts each infant’s choice of the similar puppet (black bar) or the dissimilar puppet (gray bar). The similar puppet is defined as the puppet that stated it liked the same food the infant chose during the food choice task; the dissimilar puppet is defined as the puppet that stated it disliked the same food the infant chose during the food choice task. The right graph depicts whether infants reached for the right side (black bar) or left side (gray bar) when choosing a puppet.

On at least 80% of trials, one infant (8%) chose the similar puppet and three infants (25%) chose the dissimilar puppet (see Figure 5, left graph). Eight infants (67%) chose a puppet from the same side (see Figure 5, right graph).

#### Manipulation Check

Twenty parents (83%) correctly answered all three questions and the remaining four parents (17%) correctly answered two of the three questions.

#### Infant Food Choice

In both groups, 50% of the infants selected graham crackers and 50% selected green beans. Because green beans were always presented on infants’ right side (with the two exceptions described in the Method section), we examined whether side preference correlated with food choice. In the Replication and Extension Group, among the five infants who chose the same side on at least 80% of both pre- and post-manipulation trials, three (60%) also chose the food presented on that side. Among the 18 infants from the Experimental Group and the Replication and Extension Group (during the five post-manipulation trials) who chose the same side across at least four of the five trials, seven (39%) also chose the food presented on that side.

#### Post-study Questionnaire

Parent responses to question 1 were coded based on the words and phrases listed in Table 1. Six parents (25%) correctly described the purpose of the study; four were from the Replication and Extension Group. Twelve parents (50%) correctly identified the key dependent and independent variables (e.g., preference, similarity), but did not accurately describe the purpose of the puppet show; of these parents, five were from the Replication and Extension Group. Among the 18 infants whose parents correctly identified the nature of the puppet show or the key variables, five (38%) chose the similar puppet on the first trial (*p* = 0.0963, binomial test, two-tailed).

All 24 parents correctly identified their infant’s food choice; 8 parents (33%) correctly identified the similar puppet, 6 parents (25%) incorrectly identified the similar puppet, and 10 parents (42%) reported they did not know which puppet was the similar puppet.

In terms of infants’ history with the two foods, 5 parents (21%) reported their infant “often” consumed green beans, 6 parents (25%) reported their infants “sometimes” ate green beans, and 13 parents (54%) reported their infant “rarely” or “never” consumed green beans. No parents reported their infant “often” consumed graham crackers, 5 (21%) reported their infant “sometimes” ate graham crackers, and 19 parents (79%) reported their infant “rarely” or “never” consumed graham crackers. Of the 13 infants whose parents reported they rarely or never consumed green beans, 7 (54%) chose green beans, and of the 19 infants whose parents reported their infant rarely or never consumed graham crackers, 9 (47%) chose graham crackers.

### Summary

Overall, a total of six infants (25%) chose the similar puppet on the first trial. In terms of side, 7 of the infants (58%) in the Replication and Extension Group chose a puppet on the left side and 8 (67%) in the Experimental Group chose a puppet on the right side. Across repeated trials, 6 infants (25%) chose the same puppet (12.5% chose the similar puppet) and 18 infants (75%) chose a puppet on the same side. Green beans were chosen by 50% of the infants. Thirteen parents (54%) reported their infant “rarely” or “never” consumed green beans and 19 parents (79%) reported their infant “rarely” or “never” consumed graham crackers, yet nearly half of these infants chose the food their parent reported they rarely or never consumed.

## General Discussion

The purpose of the current study was to replicate and extend the single-choice puppet paradigm commonly used to examine infants’ social evaluations and preferences for similar others. This method has been reported in many studies (e.g., Martin and Olson, 2015; Holvoet et al., 2016; Margoni and Surian, 2018; Van de Vondervoort and Hamlin, 2018) and is often cited as evidence for infants’ innate tendencies. We based our replication on Mahajan and Wynn (2012), who used the single-choice puppet paradigm to examine infants’ tendency to prefer similar others. We then extended the methods by including (1) within-subject repeated measures and (2) an experimental manipulation of a plausible demand characteristic. Overall, our results from Experiment 1 and Experiment 2 suggest that on the first-choice trial, infants were not more likely to choose the similar puppet, and across repeated trials more infants chose a puppet from the same side than the puppet similar to themselves. Manipulation of the demand characteristic, parent knowledge of infants’ food choice (Experiment 1), and parents’ reading about the purpose of the study (Experiment 2), appeared to have little effect on infants’ puppet choices, with one exception, described below.

Across both experiments (*N* = 44), 39% of the infants chose the similar puppet on the first-choice trial. Interestingly, 83% of the infants whose parents read an informational flyer about the study prior to their infants making their first puppet choice (Experiment 2) chose the dissimilar puppet on the first trial. It is unclear whether the demand characteristic manipulation played a role in infants’ choosing the dissimilar puppet; infants whose parents read the article after infants had made three to five choices did not choose the dissimilar puppet more often across the remaining five choice trials. Moreover, more infants chose a puppet based on side rather than the similarity of the puppet; across both experiments, 55% of the infants chose a puppet on the right side on the first-choice trial.

Perhaps more striking were the patterns that emerged in the within-subject repeated measures. Across both experiments, on at least 80% trials, 15% of the infants chose the similar puppet but 64% of the infants chose a puppet from the same side. These findings are consistent with Nighbor et al. (2017) who found that 50% of the infants chose a puppet from the same side on at least four of the five trials. Salvadori et al. (2015) also noted that 71% of the infants on the first (and only) trial chose a puppet from the right side, compared to 50% who chose the prosocial puppet. Moreover, because the puppet choice paradigm makes use of two identical puppets that differ only by the color of the shirts they are wearing findings from Diedrich et al. (2001) suggest this tactic may inadvertently encourage infant side perseveration rather than assess infants’ preferences for a particular puppet.

Another central variable warranting closer analysis is infant food selection. Per the current methodology, all infants were asked to choose either graham crackers or green beans. In many studies, infants’ selection of the similar puppet is predicated on the assumption that the infant is choosing a food based on a preference or liking of that food (e.g., Hamlin and Wynn, 2012; Mahajan and Wynn, 2012), although infants’ history with these foods is rarely assessed. In Experiment 2, we did inquire about infants’ history with the foods. Surprisingly, a majority of the parents reported their infants rarely or never ate green beans (54%), yet over half of these infants chose green beans during the food choice, and (79%) of parents reported their infants rarely or never ate graham crackers, yet just under half of these infants chose graham crackers. Thus, familiarity with the food appears not to have affected the likelihood of the infants selecting a particular food. If infants have no history with a food, it is unlikely that they have developed or can demonstrate a preference for that food (e.g., Paroche et al., 2017). By extension, if they have no clear food preference, how can they choose a puppet with a preference similar to theirs (i.e., the “similar” puppet)? Without a similar puppet, the conclusion that infants demonstrate a preference for similar others is speculative at best; infants’ preference or liking ought to be explicitly assessed. As several studies have demonstrated, it is not unreasonable to employ procedures in which infants sample the foods and a determination of their preference is based on observed behavior (e.g., Repacholi and Gopnik, 1997; Hamlin and Wynn, 2012; Ruffman et al., 2018; Zonneveld et al., 2018), rather than a single reach toward a potentially unfamiliar food.

## Limitations

Results of the current study must be considered within the context of several potential limitations. First, although we attempted to replicate Mahajan and Wynn’s (2012) methodology exactly, we did deviate somewhat from their protocol. Mahajan and Wynn (2012) conducted all of their sessions in the same laboratory setting, whereas 12 of 20 sessions in Experiment 1 and 22 sessions in Experiment 2 were conducted in participants’ homes because many participants were unable to travel to the lab. Every attempt was made to control for distracting stimuli and events (e.g., removed any visible toys, turned off electronics, asked parents to turn off phones, etc.) and most studies employing the single-choice puppet paradigm report some modifications in the procedure. When we examined infant choice by study location in Experiment 1, the data showed that 75% of the infants tested in the lab (*n* = 12) chose the similar puppet and the green beans and 62.5% chose a puppet from the left side on the first trial. Whereas, among infants in Experiment 1 tested in their homes (*n* = 8), 42% chose the similar puppet, 58% chose the green beans, and 50% chose the puppet on the left side on the first trial. These results suggest that small nuances or changes in study location, despite the set-up, puppet show, and other parameters remaining the same, may influence infants’ behavior. Similar to failed replications examining infant social evaluations using looking times (Holvoet et al., 2017), our results highlight the importance of clearly identifying and documenting the parameters of the puppet paradigm which are necessary for infants to reliably demonstrate preferences for similar others (Eason et al., 2017).

Second, our sample size was small, though it did not differ appreciably from similar studies (Margoni and Surian, 2018), and the age range was somewhat broader than is typically reported in similar studies. The current study included a total of 44 infant/parent dyads (20 in Experiment 1 and 24 in Experiment 2), and these numbers were determined prior to the start of data collection. Although recommended (Simmons et al., 2011), a description for pre-determining sample size is rarely provided in published studies using the single-choice infant paradigm (e.g., Hamlin et al., 2007, 2010, 2013; Mahajan and Wynn, 2012; Gerson et al., 2017; Chae and Song, 2018; Margoni and Surian, 2018) and at times is not included as part of the study protocol (Eason et al., 2017). Small sample sizes are problematic, as they increase the likelihood of spurious findings (Oakes, 2017). Failure to predetermine sample size is at least as problematic, as it can lead to the intentional or unintentional practice of “p-hacking” or determining when to terminate data collection based on reaching desired results rather than on predetermined criteria (Schulz and Grimes, 2005; Simmons et al., 2011; Peterson, 2016; Oakes, 2017). We support the call for *a priori* decisions about sample size (e.g., Simmons et al., 2011; Simonsohn, 2015; Eason et al., 2017) and use of power analyses to help determine this sample size. We also support the use of within-subject repeated measures to identify meaningful within and between-subject differences through differentiated data patterns (Loftus, 1993). Within-subject repeated measures can address such problems as spurious findings and p-hacking, as well as questions of parental influence on infants’ choices, without much in the way of additional effort or cost on the part of the researcher (e.g., Eason et al., 2017).

Third, an argument could be made that infants’ first choice represents their true preference and that use of repeated measures confuses infants. We required infants to make up to 10 choices. Presumably, innate or strong preferences ought to be a relatively reliable phenomenon. There currently exist well-established methods for assessing preferences across within-subject repeated trials, even among individuals with limited language (e.g., Kang et al., 2013). For example, when multiple items are available across multiple trials, the first item chosen by a child is only their most preferred or most often chosen item about half the time (Rapp et al., 2010). However, it is possible that our method of presenting repeated measures in rapid succession pulled more for side perseveration and less for actual preference. We recommend this be examined in future research by, for example, spacing out the choice trials by 1–5 min, perhaps with puppet shows preceding each choice, or by having the same infants return for additional assessments. Repeated choice trials for all dependent measures (e.g., food and puppets) might increase the accuracy of the infants’ preferences if they exist and would strengthen our understanding of the reliability and robustness of the phenomenon under investigation (Loftus, 1993; Dahl et al., 2013; Martin and Olson, 2015; Smith and Little, 2018).

## Summary and Future Directions

The cornerstones of science are independent replication and experimental control (Sidman, 1960; Ioannidis, 2005; Rosenthal, 2009; Simmons et al., 2011; Novella, 2012; Open Science Collaboration, 2015). Independent research teams (e.g., Scarf et al., 2012; Cowell and Decety, 2015; Salvadori et al., 2015; Holvoet et al., 2017; Nighbor et al., 2017) have been unable to replicate the results showing infants’ preference for prosocial others. To further our understanding of infants’ tendencies, we support the recommendation made by others, including publishing null finding (Franco et al., 2014; Margoni and Surian, 2018), standardizing data collection and reporting methods (Simmons et al., 2011; Martin and Olson, 2015; Holvoet et al., 2016; Peterson, 2016; Eason et al., 2017; Oakes, 2017), and examining individual differences (Martin and Olson, 2015), for example, by employing single subject designs with repeated measures (e.g., Loftus, 1993; Ruffman et al., 2018; Smith and Little, 2018).

## Data Availability Statement

The raw data supporting the conclusions of this manuscript will be made available by the authors, without undue reservation, to any qualified researcher.

## Author Contributions

AC-K and KB contributed equally to this work and each wrote a first draft of the manuscript. This study is based on theses submitted by AC-K and KB in partial fulfillment of the MA degree requirements at University of the Pacific. AC-K, KB, CK, MN, and HS contributed to the conception and design of the study, contributed to the manuscript revision, and read and approved the submitted version. AC-K, KB, and CK performed the statistical analyses and graphed the data. CK wrote sections of the manuscript.

### Conflict of Interest

The authors declare that the research was conducted in the absence of any commercial or financial relationships that could be construed as a potential conflict of interest.

## Footnotes

^1^The other common methodology uses infants’ looking time as the dependent measure, but discussion of this method is outside the scope of this paper.

^2^https://campuspress.yale.edu/infantlab/media/

^3^Mahajan and Wynn (2012) initially compared two groups of infants, one group choosing between graham crackers/Cheerios™ and another choosing between graham crackers/green beans. Because they found no between- group differences, we decided to use green beans, as they are more visibly distinct from graham crackers than Cheerios™.

^4^http://campuspress.yale.edu/infantlab/

^5^http://campuspress.yale.edu/infantlab/about-us/faq/

^6^http://campuspress.yale.edu/infantlab/our-studies/
